# Supplementary material for: Hybrid assembly of polymeric nanofiber network for robust and electronically conductive hydrogels
Source: Nat Commun. 2023 Feb 10;14:759. doi: 10.1038/s41467-023-36438-8 (PMC9918487; doi:10.1038/s41467-023-36438-8)
Supplement: Supplementary file 1 — Supplementary Information [file 41467_2023_36438_MOESM1_ESM.pdf]

Supplementary Information

Hybrid Assembly of Polymeric Nanofiber Network for Robust and Electronically Conductive Hydrogels

Huimin He[1,4], Hao Li[1,4], Aoyang Pu[2], Wenxiu Li[2], Kiwon Ban[2], and Lizhi Xu[1,3]✉

[1]Department of Mechanical Engineering, The University of Hong Kong, Hong Kong SAR, China.

[2]Department of Biomedical Sciences, City University of Hong Kong, Hong Kong SAR, China.

[3]Advanced Biomedical Instrumentation Centre Limited, Hong Kong SAR, China.

[4]These authors contributed equally: Huimin He and Hao Li.

✉e-Email: xulizhi@hku.hk (L.X)

Supplementary Figures 1-17

Supplementary Table 1

Supplementary Videos 1-2

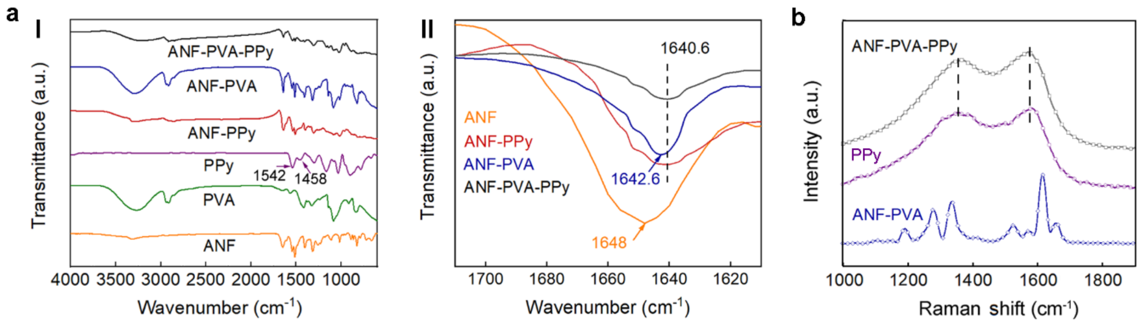

Supplementary Fig. 1 | Chemical structures characterized by FTIR and Raman spectroscopy. a, FTIR spectra (I) of ANF, PVA, PPy, ANF-PPy, ANF-PVA, and ANF-PVA-PPy. Magnified plot (II) showing spectra of aramid C=O stretching vibration in ANF, ANF-PVA, ANF-PPy and ANF-PVA-PPy. For ANF-PPy and ANF-PVA-PPy, characteristic peaks for C═C and ═C−N symmetric and asymmetric ring stretching were measured at 1542 and 1458 cm^{-1} respectively, demonstrating the successful integration of PPy. For pure ANF, the aramid C═O stretching band was peaked at 1648 cm^{-1}. ANF-PVA has a red-shift of this peak to 1642.6 cm^{-1}, reflecting the effects of hydrogen bonding between ANF and PVA. A larger scale of red-shift to 1640.6 cm^{-1} was found in ANF-PPy composites, which indicates hydrogen bonding between ANF and PPy as well. **b,** Raman spectra of ANF-PVA, PPy, and ANF-PVA-PPy indicate the incorporation of PPy in the composites. Source data are provided as a Source Data file.

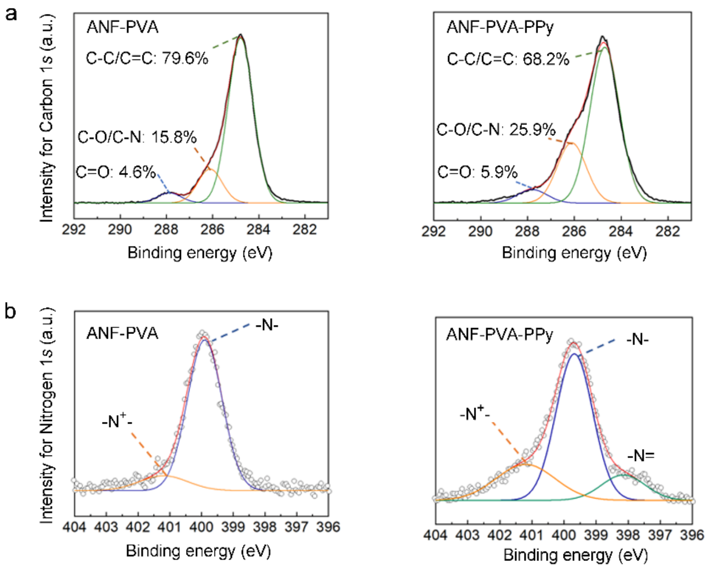

Supplementary Fig. 2 | XPS spectra of ANF-PVA and ANF-PVA-PPy. a, C 1*s* spectra of ANF-PVA and ANF-PVA-PPy. **b,** N 1*s* spectra of ANF-PVA and ANF-PVA-PPy. The C 1*s* spectra have 3 peaks at binding energies of 284.8, 286.1, and 287.9 eV, corresponding to C−C/C═C, C−O/C−N, and C═O, respectively. The presence of PPy is indicated by the increase of peak intensity at 286.1 eV. For N 1*s* spectra, 3 peaks at 398.1, 399.6, and 401.2 eV represent −N═, −N−, and $-NH^{+}-$ respectively. The emerging peak at 398.1 eV corresponding to −N═ further confirms the incorporation of PPy into ANF-PVA-PPy. The significant increase of the peak intensity at 401.2 eV also demonstrates peroxidation of PPy in the presence of excess oxidant of $FeCl_3$. Source data are provided as a Source Data file.

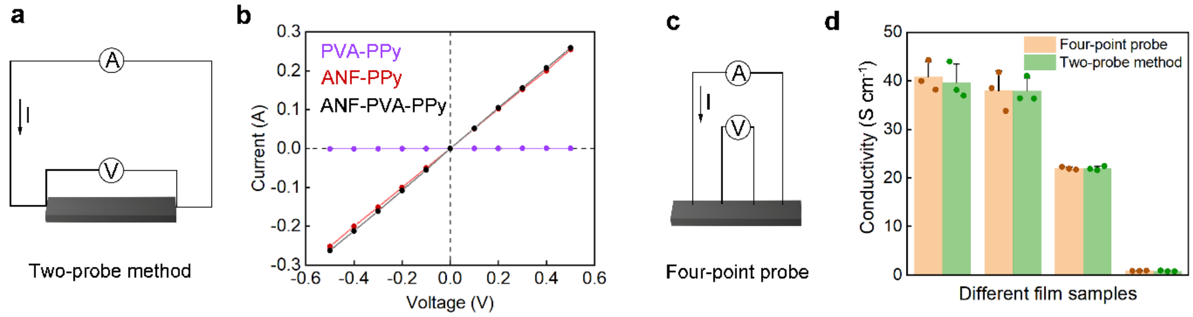

Supplementary Fig. 3 | Measurement of electrical conductivity. a, A schematic of conductivity measured by two-probe method. **b,** Current measured with bias voltage from -0.5 to 0.5 V by two-probe method, showing linear responses of CNHs. **c,** A schematic of conductivity measured by four-point probe. **d,** Conductivities measured by the two different methods are consistent, based on various ANF-PVA-PPy samples with different levels of conductivity. Data in **d** represent their means ± SDs from n=3 independent samples. Source data are provided as a Source Data file.

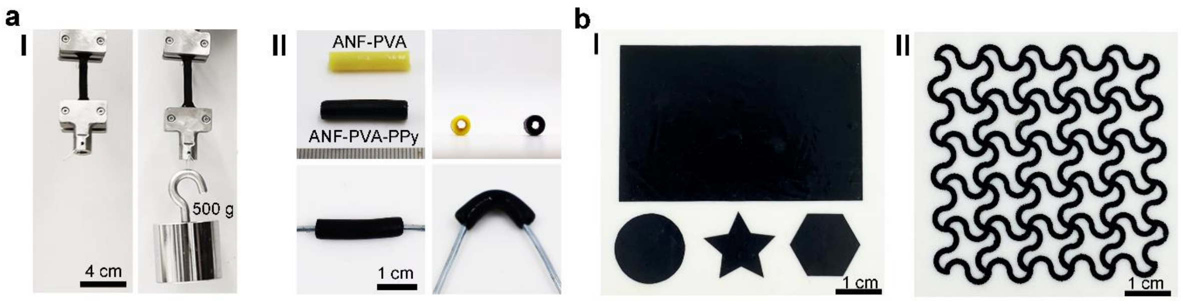

Supplementary Fig. 4 | Photographs of bulk and film ANF-PVA-PPy. a, Photographs of a strip sample under tension (I) and hollow tubes which can withstand bending (II). **b,** Photographs of films with various shapes (I) and patterned by infrared laser machining (II).

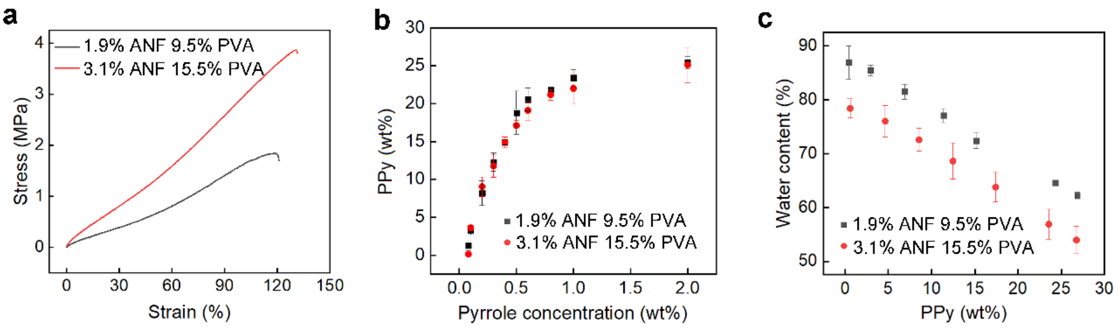

Supplementary Fig. 5 | Control of PPy content for ANF-PVA-PPy. a, Tensile stress-strain curves for 1.9% ANF 9.5% PVA and 3.1% ANF 15.5% PVA hydrogels. **b,** The PPy content in ANF-PVA-PPy hydrogels is tunable with various pyrrole concentrations during polymerization. The PPy content raised with the increase of pyrrole concentration until 1 wt% and then reached a plateau. **c,** The water content of conductive hydrogels. Both the CNHs prepared from 1.9% ANF 9.5% PVA and 3.1% ANF 15.5% PVA matrices demonstrate linear decrease in water content with increasing PPy content. Data in **b** and **c** represent their means ± SDs from n=3 independent samples. Source data are provided as a Source Data file.

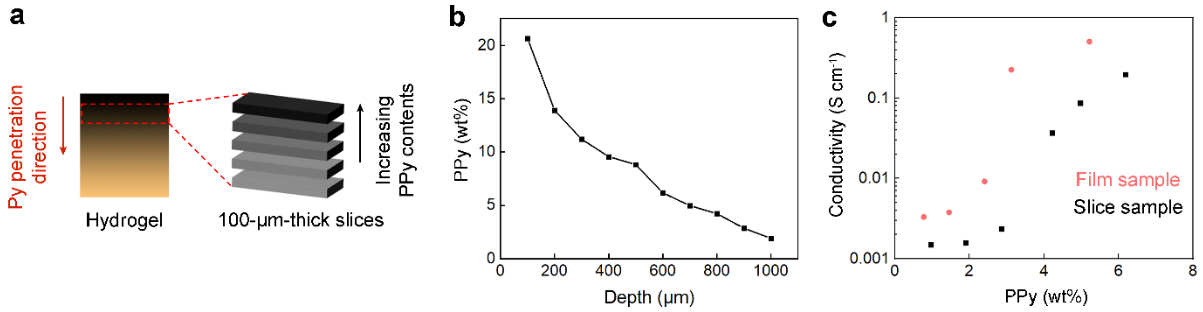

Supplementary Fig. 6 | PPy distribution within bulk hydrogels. a, Schematics of slicing a bulk ANF-PVA-PPy hydrogel with 100-μm-thick layers. **b,** PPy content of each layer as a function of depth from the surface. The sample was obtained from Py (0.4 M), $FeCl_3$ (0.48 M), and 3.1% ANF 15.5% PVA matrix (18 mm in diameter and 8 mm in height) during the synthesis. **c,** Electrical conductivity as a function of PPy content for CNHs with 3.1% ANF 15.5% PVA matrix, obtained from both slice samples and film samples. The results show consistency of measured threshold for electrical percolation. The lower absolute values of conductivity for slice samples may be attributed to the structural defects introduced during the mechanical cryo-sectioning process. Source data are provided as a Source Data file.

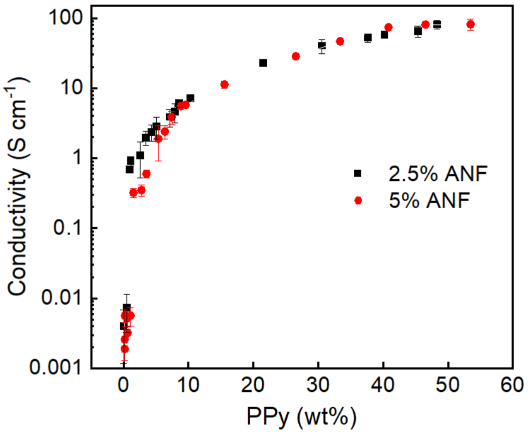

Supplementary Fig. 7 | Effects of PPy contents on the conductivity of ANF-PPy hydrogels. The electrical conductivity of ANF-PPy with sparser nanofiber template (2.5% ANF) is higher than that of 5% ANF before the PPy content reaches 10 wt%. Then samples showed similar electrical conductivity under same PPy contents. Data represent their means ± SDs from n=3 independent samples. Source data are provided as a Source Data file.

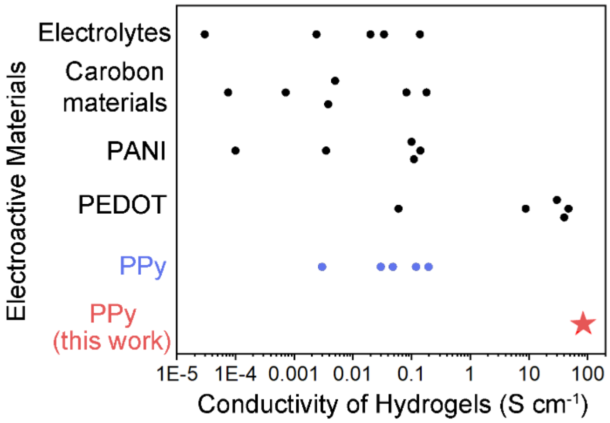

Supplementary Fig. 8 | Comparation of conductivities between CNHs and other reported conductive hydrogels. Data points were categorized based on the electroactive components in the hydrogels. More details are shown in Supplementary Table 1. Source data are provided as a Source Data file.

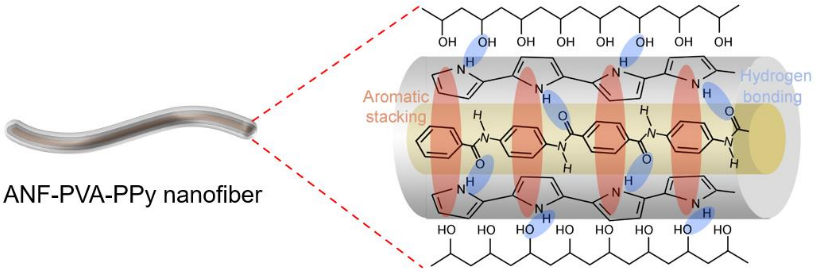

Supplementary Fig. 9 | Schematic illustration of possible intermolecular interactions in ANF-PVA-PPy. The aromatic rings and amide groups on ANFs afford attractions to PPy via π-π stacking and hydrogen bonding. Hydrogen bonding is also possible between PPy and PVA, as well as PVA and ANF.

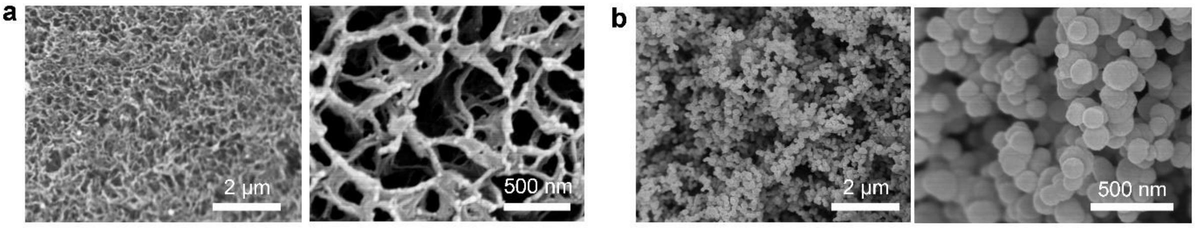

Supplementary Fig. 10 | SEM images of PPy distributed in various matrices. ANF-PPy (**a**) exhibited fibrillar networks while PPy formed spherical particles randomly distributed in the PVA matrix (**b**). The SEM images are based on cross-sections of hydrogel samples.

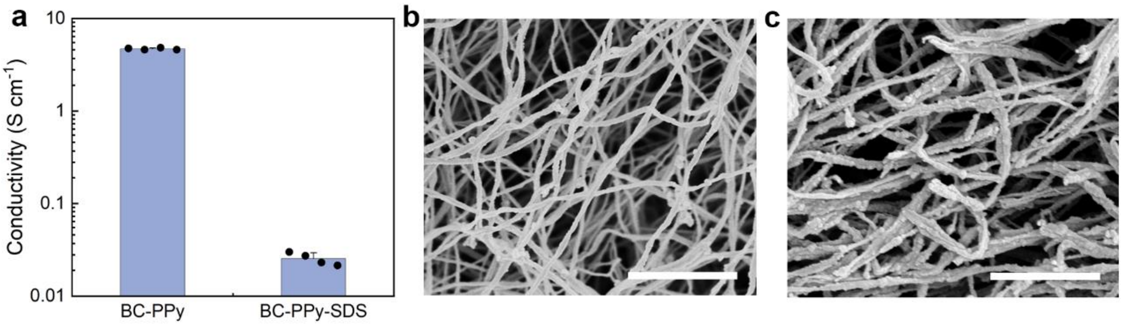

Supplementary Fig. 11 | Templating effect of bacterial cellulose (BC) hydrogels. a, Comparison of electrical conductivities between BC-PPy and BC-PPy-SDS hydrogels prepared under similar polymerization conditions.**b-c**, SEM images of BC (**b**) and BC-PPy (**c**). The PPy polymerized along the nanofibers indicates the templating effect of BCs. Scale bars: 1μm. Data in **a** represent their means ± SDs from n=4 independent samples. Source data are provided as a Source Data file.

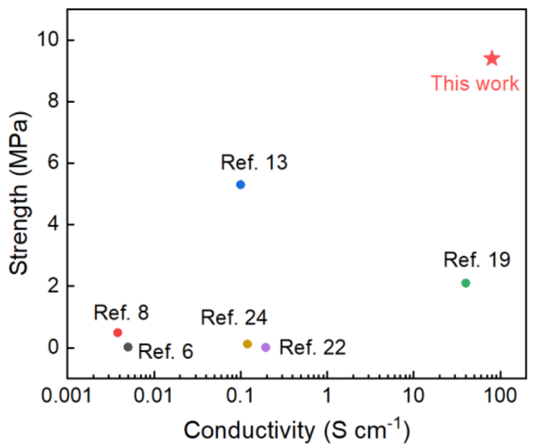

Supplementary Fig. 12 | Comparison of conductivities and mechanical strengths between CNHs and other reported conductive hydrogels. CNH exhibits outstanding electronic conductivity and mechanical strength. Source data are provided as a Source Data file.

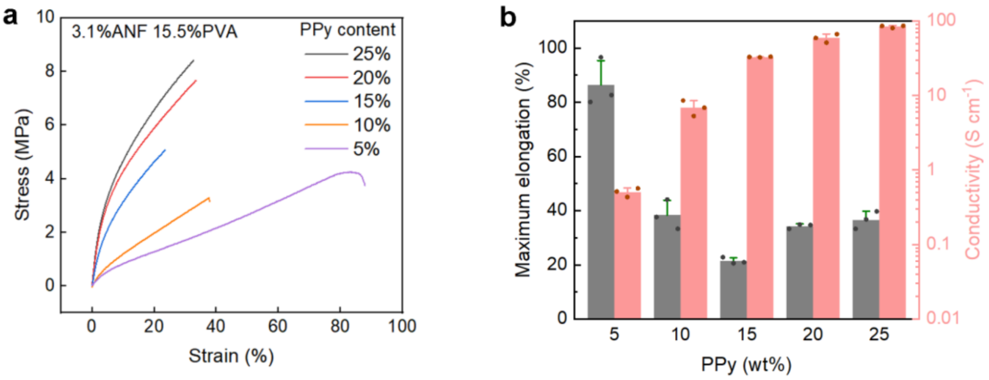

Supplementary Fig. 13 | Tensile behaviors of CNHs with various PPy contents based on matrices with 3.1% ANF 15.5% PVA. a, Stress-strain curves. **b**, Maximum elongation and conductivity of various samples. Data in **b** represent their means ± SDs from n=3 independent samples. Source data are provided as a Source Data file.

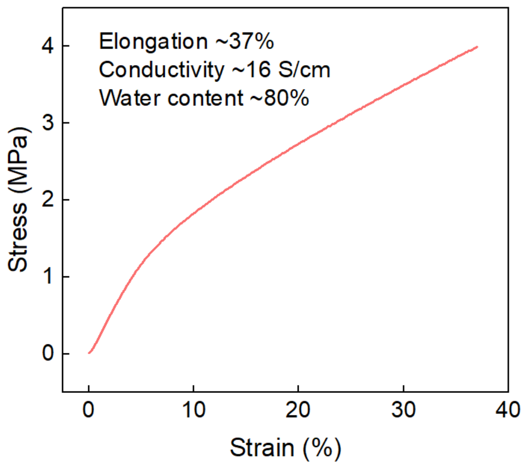

Supplementary Fig. 14 | Tensile stress-strain curve for a representative CNH. In an optimized configuration, the hydrogel shows a stretchability of 37%, conductivity of 16 S/cm, and water content of 80%. The hydrogel was synthesized based on 1.9% ANF 9.5% PVA matrix and 0.3 wt% pyrrole, polymerized under pH 7 and 0°C for 2 h. Source data are provided as a Source Data file.

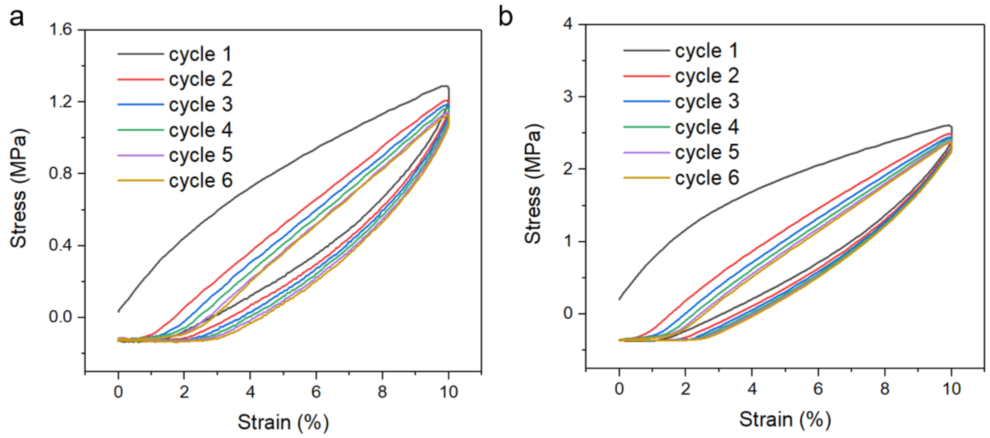

Supplementary Fig. 15 | CNHs with considerable stability under cyclic deformation. Cyclic stress-strain curves for CNHs based on 1.9% ANF, 9.5% PVA, 10% PPy **(a)** and 3.1% ANF, 15.5% PVA, 15% PPy **(b).** Source data are provided as a Source Data file.

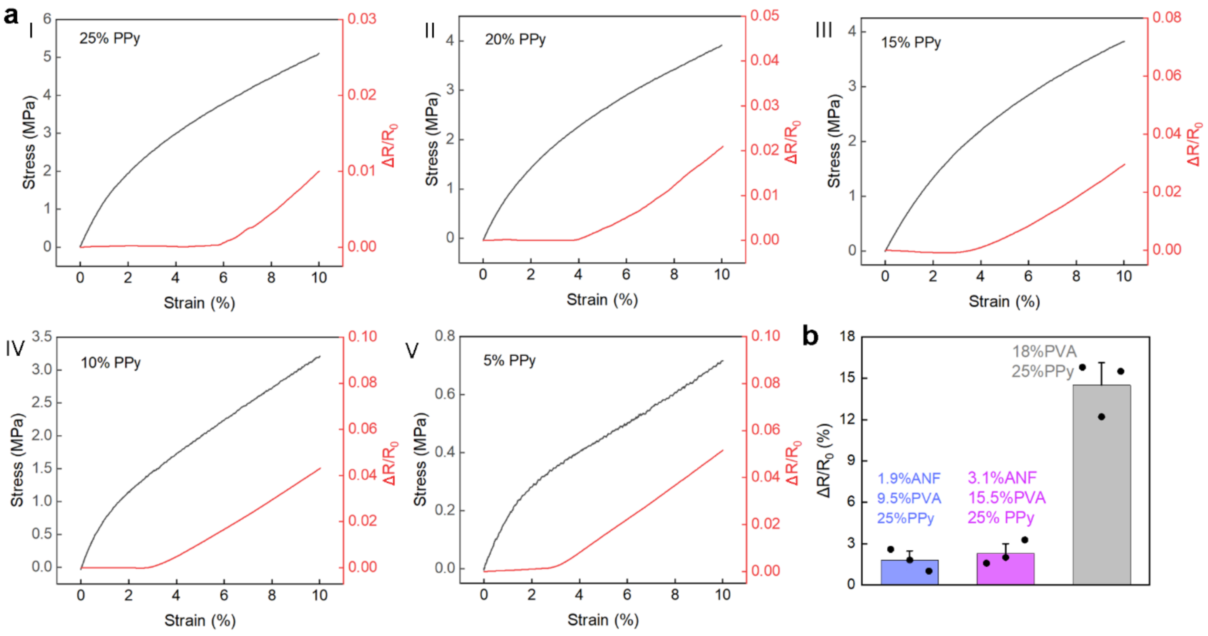

Supplementary Fig. 16 | Mechanical and electrical behaviors of CNHs under tensile deformation. a, The samples are based on 1.9% ANF 9.5% PVA matrices and incorporated with a PPy content of 25 wt% (**I**), 20 wt% (**II**), 15 wt% (**III**), 10 wt% (**IV**), and 5 wt% (**V**). The stress and change of resistance ($\Delta R/R_0$) are recorded simultaneously under imposed tensile strain, showing strain-invariant conductance within the initial range of deformation. **b,** Comparison of electrical responses of CNHs and PVA-PPy on tensile deformation of 10%. Data in **b** represent their means ± SDs from n=3 independent samples. Source data are provided as a Source Data file.

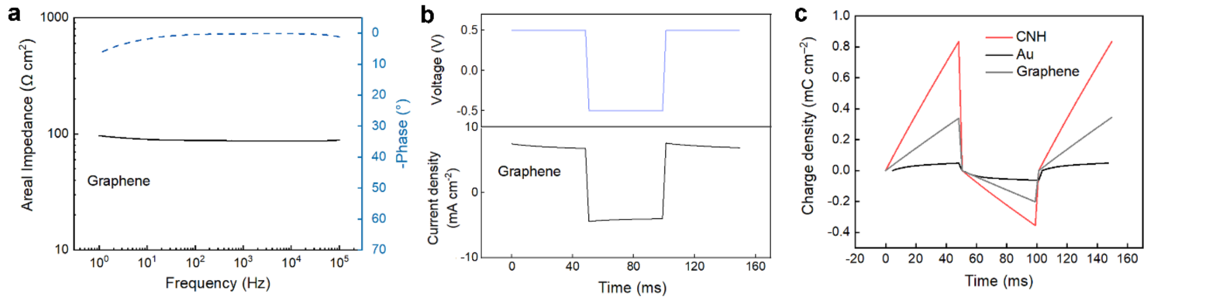

Supplementary Fig. 17 | Comparison of electrochemical properties. a, b, Electrochemical impedance spectroscopy (EIS) (**a**) and pulsed current injection curves for a graphene electrode with the voltage switching between -0.5 and 0.5 V vs. Ag/AgCl (**b**). **c,** Cyclic electrochemical current pulse injection curves of electrodes based on CNH, Au and graphene, with the voltage switching between −0.5 V and 0.5 V vs. Ag/AgCl. Source data are provided as a Source Data file.

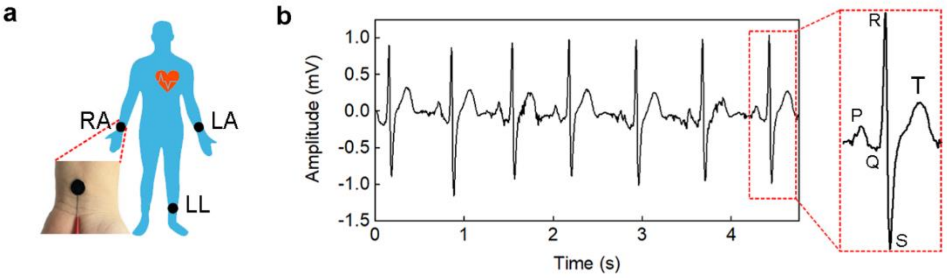

Supplementary Fig. 18 | Measurement of ECG using CNH electrodes. a, The setup for the measurement. **b,** ECG signals captured by the CNH electrodes, showing details of QRS, T and P waves. Source data are provided as a Source Data file.

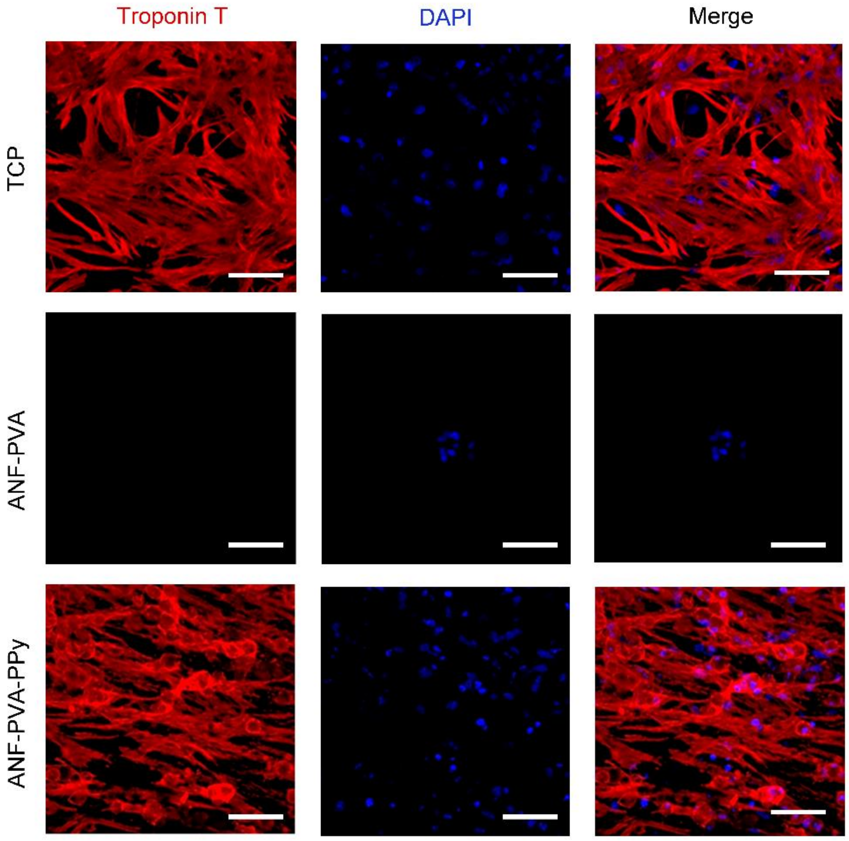

Supplementary Fig. 19 | Immunostaining of troponin (red) and nuclei (blue) of neonatal rat cardiomyocytes cultured on TCPs, ANF-PVA and ANF-PVA-PPy respectively. Cells are well attached to ANF-PVA-PPy substrates, showing good alignment and expression of cardiac-specific protein troponin T. Scale bar: 50 μm.

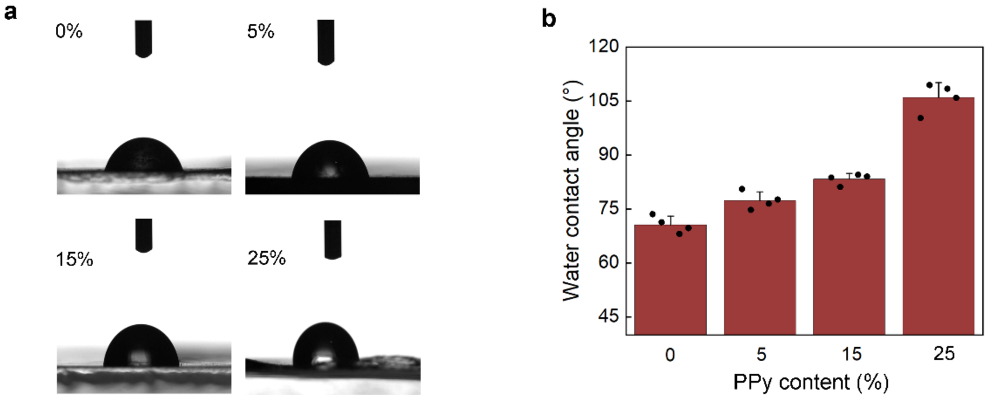

Supplementary Fig. 20 | Wettability of ANF-PVA-PPy with different PPy contents. a, photos of water droplets (3μl) on the surfaces of ANF-PVA-PPy (3.1% ANF, 15.5% PVA) with various PPy contents after 10s contact. **b,** Water contact angles as a function of PPy content. Data in **b** represent their means ± SDs from n=4 independent samples. Source data are provided as a Source Data file.

Supplementary Table 1 | Summary of conductivities of other reported electroconductive hydrogels.

Electroactive Materials		Hydrogel Matrix	Conductivity (S cm^{-1})	Ref.
Electrolytes	NaCl	Hydroxypropyl cellulose/PVA	0.034	1
	LiCl	PAAm	0.139	2
	sodium citrate	Aramid nanofiber/PVA	0.02	3
	Na_2SO_4	PEG	0.0024	4
	choline chloride	Poly α-lipoic acid	0.00003	5
Carbon materials	GO	GO	0.005	6
	graphene	PAAm	0.000075	7
	graphene	Collagen	0.0038	8
	rGO	Polydopamine/PAAm	0.18	9
	CNTs	P(AAm-AAc)	0.082	10
	CNTs	Chitosan/Gelatin	0.00072	11
PANI	PANI	Chitosan/PEG/PANI	0.0035	12
	PANI	PVA/PANI	0.1	13
	PANI	PSS/PANI	0.142	14
	PANI	Phytic acid/PANI	0.11	15
	PANI	N-fluorenylmethoxycarbonyl diphenylalanine/PANI	0.0001	16
PEDOT	PEDOT	PEDOT/PSS	8.8	17
	PEDOT	PEDOT/PSS	47.4	18
	PEDOT	PEDOT/PSS	40	19
	PEDOT	PEDOT/sulfonated lignin/PAAc	0.06	20
	PEDOT	PU/ PEDOT/PSS	30	21
PPy	PPy	Agarose	0.195	22
	PPy	Silk fibroin	0.03	23
	PPy	PAAm	0.12	24
	PPy	Cellulose nanofibers/PVA	0.048	25
	PPy	PAAm/Chitosan	0.003	26

References

1. Zhou, Y. et al. Highly stretchable, elastic, and ionic conductive hydrogel for artificial soft electronics. *Adv. Funct. Mater.* **29,** 1806220 (2019).
2. Tian, K. et al. 3D printing of transparent and conductive heterogeneous hydrogel–elastomer systems. *Adv. Mater.* **29,** 1604827 (2017).
3. He, H. et al. Elastic, conductive, and mechanically strong hydrogels from dual-cross-linked aramid nanofiber composites. *ACS Appl. Mater. Interfaces* **13,** 7539-7545 (2021).
4. Zhao, S. et al. Programmable hydrogel ionic circuits for biologically matched electronic interfaces. *Adv. Mater.* **30,** 1800598 (2018).
5. Dang, C. et al. Transparent, highly stretchable, rehealable, sensing, and fully recyclable ionic conductors fabricated by one-step polymerization based on a small biological molecule. *Adv. Funct. Mater.* **29,** 1902467 (2019).
6. Xu, Y., Sheng, K., Li, C. & Shi, G. Self-assembled graphene hydrogel via a one-step hydrothermal process. *ACS Nano* **4,** 4324-4330 (2010).
7. González-Domínguez, J. M., Martín, C., Durá, O., Merino, S. & Vázquez, E. Smart hybrid graphene hydrogels: A study of the different responses to mechanical stretching stimulus. *ACS Appl. Mater. Interfaces* **10,** 1987-1995 (2018).
8. Agarwal, G., Kumar, N. & Srivastava, A. Highly elastic, electroconductive, immunomodulatory graphene crosslinked collagen cryogel for spinal cord regeneration. *Mater. Sci. Eng. C.* **118,** 111518 (2021).
9. Han, L. et al. A mussel-inspired conductive, self-adhesive, and self-healable tough hydrogel as cell stimulators and implantable bioelectronics. *Small* **13,** 1601916 (2017).
10. Han, L. et al. Mussel-inspired adhesive and conductive hydrogel with long-lasting moisture and extreme temperature tolerance. *Adv. Funct. Mater.* **28,** 1704195 (2018).
11. Liang, Y., Zhao, X., Hu, T., Han, Y. & Guo, B. Mussel-inspired, antibacterial, conductive, antioxidant, injectable composite hydrogel wound dressing to promote the regeneration of infected skin. *J. Colloid and Interface Sci.* **556,** 514-528 (2019).
12. Zhao, X., et al. Antibacterial anti-oxidant electroactive injectable hydrogel as self-healing wound dressing with hemostasis and adhesiveness for cutaneous wound healing. *Biomaterials* **122,** 34-47 (2017).
13. Li, W., Gao, F., Wang, X., Zhang, N. & Ma, M. Strong and robust polyaniline-based supramolecular hydrogels for flexible supercapacitors. *Angew. Chem.* **128,** 9342-9347 (2016).
14. Chen, J., Peng, Q., Thundat, T. & Zeng, H. Stretchable, injectable, and self-healing conductive hydrogel enabled by multiple hydrogen bonding toward wearable electronics. *Chem. Mater.* **31,** 4553-4563 (2019).
15. Pan, L. et al. Hierarchical nanostructured conducting polymer hydrogel with high electrochemical activity. *PNAS* **109,** 9287-9292 (2012).
16. Chakraborty, P. et al. A self-healing, all-organic, conducting, composite peptide hydrogel as pressure sensor and electrogenic cell soft substrate. *ACS Nano* **13,** 163-175 (2018).
17. Yao, B. et al. Ultrahigh-conductivity polymer hydrogels with arbitrary structures. *Adv. Mater.* **29,** 1700974 (2017).
18. Liu, Y. et al. Soft and elastic hydrogel-based microelectronics for localized low-voltage neuromodulation. *Nat. Biomed. Eng.* **3,** 58-68 (2019).
19. Lu, B. et al. Pure PEDOT:PSS hydrogels. *Nat. Commun.* **10,** 1043 (2019).
20. Wang, Q. et al. Biocompatible, self-wrinkled, antifreezing and stretchable hydrogel-based wearable sensor with PEDOT: sulfonated lignin as conductive materials. *J. Chem. Eng.* **370,** 1039-1047 (2019).

21. Inoue, A., Yuk, H., Lu, B. & Zhao, X. Strong adhesion of wet conducting polymers on diverse substrates. *Sci. Adv.* **6,** eaay5394 (2020).
22. Hur, J. et al. Polypyrrole/agarose-based electronically conductive and reversibly restorable hydrogel. *ACS Nano* **8,** 10066-10076 (2014).
23. Mo, F. et al. A Self-Healing Crease-Free Supramolecular All-Polymer Supercapacitor. *Adv. Sci.* **8,** 2100072 (2021).
24. Han, L. et al. Transparent, adhesive, and conductive hydrogel for soft bioelectronics based on light-transmitting polydopamine-doped polypyrrole nanofibrils. *Chem. Mater.* **30,** 5561-5572 (2018).
25. Ding, Q. et al. Nanocellulose-mediated electroconductive self-healing hydrogels with high strength, plasticity, viscoelasticity, stretchability, and biocompatibility toward multifunctional applications. *ACS Appl. Mater. Interfaces* **10,** 27987-28002 (2018).
26. Gan, D. et al. Conductive and tough hydrogels based on biopolymer molecular templates for controlling in situ formation of polypyrrole nanorods. *ACS Appl. Mater. Interfaces* **10,** 36218-36228 (2018).
