# Supplementary material for: Hybrid assembly of polymeric nanofiber network for robust and electronically conductive hydrogels
Source: Nat Commun. 2023 Feb 10;14:759. doi: 10.1038/s41467-023-36438-8 (PMC9918487; doi:10.1038/s41467-023-36438-8)
Supplement: Supplementary file 3 — Description of Additional Supplementary Files [file 41467_2023_36438_MOESM3_ESM.pdf]

Description for Additional Supplementary Files

Supplementary video 1: Calcium transients in cardiomyocytes cultured on TCP and CNH respectively.

Supplementary video 2: Contraction of cardiomyocytes (labelled with CellTracker CM-Dil dye) cultured on TCP, ANF-PVA, and CNH respectively.
