# Supplementary material for: Hybrid assembly of polymeric nanofiber network for robust and electronically conductive hydrogels
Source: Nat Commun. 2023 Feb 10;14:759. doi: 10.1038/s41467-023-36438-8 (PMC9918487; doi:10.1038/s41467-023-36438-8)
Supplement: Supplementary file 6 — Reporting Summary [file 41467_2023_36438_MOESM6_ESM.pdf]

nature portfolio

Corresponding author(s): Lizhi Xu

Last updated by author(s): Jan 20, 2023

Reporting Summary

Nature Portfolio wishes to improve the reproducibility of the work that we publish. This form provides structure for consistency and transparency in reporting. For further information on Nature Portfolio policies, see our Editorial Policies and the Editorial Policy Checklist.

Statistics

For all statistical analyses, confirm that the following items are present in the figure legend, table legend, main text, or Methods section.

n/a	Confirmed	
☐	☒	The exact sample size (*n*) for each experimental group/condition, given as a discrete number and unit of measurement
☐	☒	A statement on whether measurements were taken from distinct samples or whether the same sample was measured repeatedly
☒	☐	The statistical test(s) used AND whether they are one- or two-sided<br>*Only common tests should be described solely by name; describe more complex techniques in the Methods section.*
☒	☐	A description of all covariates tested
☒	☐	A description of any assumptions or corrections, such as tests of normality and adjustment for multiple comparisons
☐	☒	A full description of the statistical parameters including central tendency (e.g. means) or other basic estimates (e.g. regression coefficient) AND variation (e.g. standard deviation) or associated estimates of uncertainty (e.g. confidence intervals)
☒	☐	For null hypothesis testing, the test statistic (e.g. *F*, *t*, *r*) with confidence intervals, effect sizes, degrees of freedom and *P* value noted<br>*Give P values as exact values whenever suitable.*
☒	☐	For Bayesian analysis, information on the choice of priors and Markov chain Monte Carlo settings
☒	☐	For hierarchical and complex designs, identification of the appropriate level for tests and full reporting of outcomes
☒	☐	Estimates of effect sizes (e.g. Cohen's *d*, Pearson's *r*), indicating how they were calculated

Our web collection on statistics for biologists contains articles on many of the points above.

Software and code

Policy information about availability of computer code

Data collection	Electrical conductivity was measured by two-probe method (Keithley 2450) or a standard four-point probe (HPS2661, Helpass electronic technologies); Mechanical tension was performed on a tensile-compressive tester (Zwick Roell); Electrochemical tests were carried on by a electrochemical workstation (PGSTAT302N, Metrohm Autolab); The electrophysiological signals were recorded by a commercial data acquisition system (PowerLab T26, AD Instruments). Videos were analyzed using ImageJ2 software for Figure 5b,c.
Data analysis	The data was analysed by origin software (orignlab 8.5) and the illustrations were made by adobe illustrator 2019 software.

For manuscripts utilizing custom algorithms or software that are central to the research but not yet described in published literature, software must be made available to editors and reviewers. We strongly encourage code deposition in a community repository (e.g. GitHub). See the Nature Portfolio guidelines for submitting code & software for further information.

Data

Policy information about availability of data

All manuscripts must include a data availability statement. This statement should provide the following information, where applicable:

- Accession codes, unique identifiers, or web links for publicly available datasets
- A description of any restrictions on data availability
- For clinical datasets or third party data, please ensure that the statement adheres to our policy

The data supporting the findings of this study are available within the article and its Supplementary Information as well as Source Data. Source data are provided with this paper.

Human research participants

Policy information about studies involving human research participants and Sex and Gender in Research.

Reporting on sex and gender	This information has not been collected.
Population characteristics	31-year-old and healthy man.
Recruitment	The participant is the first author. The ability of samples to detect physiological signals would not change with selected objects. Self-selection bias will not impact results.
Ethics oversight	The Human Research Ethics Committee, The University of Hong Kong.

Note that full information on the approval of the study protocol must also be provided in the manuscript.

Field-specific reporting

Please select the one below that is the best fit for your research. If you are not sure, read the appropriate sections before making your selection.

[x] Life sciences [ ] Behavioural & social sciences [ ] Ecological, evolutionary & environmental sciences

For a reference copy of the document with all sections, see nature.com/documents/nr-reporting-summary-flat.pdf

Life sciences study design

All studies must disclose on these points even when the disclosure is negative.

Sample size	No statistical method was used to predetermine sample size. Sample sizes were based on those used in previous and preliminary studies from our lab which allow for statically valid comparisons. The sample size is four.
Data exclusions	No data was excluded.
Replication	Similar results were obtained in four independent experiments. All attempts at replication were successful.
Randomization	Throughout the cellular testing of neonatal rat cardiomyocytes (NRCMs), cells were randomized into groups of being seeded on different materials.
Blinding	No blinding was performed throughout experiments in this study. All data collected was quantifiable and blinding would not change any bias in data collected.

Reporting for specific materials, systems and methods

We require information from authors about some types of materials, experimental systems and methods used in many studies. Here, indicate whether each material, system or method listed is relevant to your study. If you are not sure if a list item applies to your research, read the appropriate section before selecting a response.

Materials & experimental systems

n/a	Involved in the study
☐	☒ Antibodies
☒	☐ Eukaryotic cell lines
☒	☐ Palaeontology and archaeology
☒	☐ Animals and other organisms
☒	☐ Clinical data
☒	☐ Dual use research of concern

Methods

n/a	Involved in the study
☒	☐ ChIP-seq
☒	☐ Flow cytometry
☒	☐ MRI-based neuroimaging

Antibodies

Antibodies used

Anti-cardiac Troponin T (Invitrogen MS-295-P1); Anti-α-Actinin (Sarcomeric) (Sigma-Aldrich A7811); Goat anti-Mouse IgG (H+L) Cross-Adsorbed Secondary Antibody, Alexa Fluor™ 568 (Invitrogen A-11004); Goat anti-Rabbit IgG (H+L) Cross-Adsorbed Secondary Antibody, Alexa Fluor™ 488 (Invitrogen A-11008)

Validation

Anti-α-Actinin (Sarcomeric) (Sigma-Aldrich A7811) has been validated for use in immunohistochemistry and western blotting in chick cardiomyocytes and rat heart samples ; Anti-Cardiac Troponin T(Invitrogen MA5-12960) antibody has been validated by Invitrogen by demonstrating immunofluorescent analysis of Troponin T Cardiac isoform in cultured primary cardiomyocytes and rat heart tissues.
